# Seasonality of childhood tuberculosis cases in Kampala, Uganda, 2010-2015

**DOI:** 10.1371/journal.pone.0214555

**Published:** 2019-04-09

**Authors:** Devan Jaganath, Eric Wobudeya, Moorine Penninah Sekadde, Betty Nsangi, Heather Haq, Adithya Cattamanchi

**Affiliations:** 1 Division of Pediatric Infectious Diseases, University of California, San Francisco, San Francisco, United States of America; 2 Directorate of Pediatrics and Child Health, Mulago National Referral Hospital, Kampala, Uganda; 3 National Tuberculosis and Leprosy Programme (NTLP), Kampala, Uganda; 4 USAID RHITES-EC, University Research Co. LLC, Kampala, Uganda; 5 Department of Pediatrics, Baylor College of Medicine, Houston, Texas, United States of America; 6 Division of Pulmonology and Critical Care Medicine, University of California, San Francisco, San Francisco, United States of America; 7 Center for Vulnerable Populations, Department of Medicine, University of California, San Francisco, San Francisco, United States of America; 8 Curry International Tuberculosis Center, University of California, San Francisco, San Francisco, United States of America; Columbia University, UNITED STATES

## Abstract

**Background:**

Seasonality in tuberculosis (TB) has been described, especially in children. However, few studies have assessed seasonality of TB in the equatorial region, and none in children.

**Objectives:**

To assess for seasonality of childhood TB cases in Kampala, Uganda, and determine the role of temperature, rainfall patterns, and influenza cases on TB diagnoses.

**Methods:**

We retrospectively analyzed demographic and clinical data of children (under 15 years) diagnosed with TB at a pediatric TB clinic in Kampala, Uganda from 2010 to 2015. We performed decomposition analysis of the monthly case time series to assess seasonality. We compared monthly mean plots and performed Poisson regression to assess any association between TB diagnoses and temperature, rainfall, and influenza.

**Results:**

Of the 713 childhood TB cases diagnosed at the clinic, 609 (85%) were clinically diagnosed and 492 (69%) were pulmonary cases. There were minimal monthly variations in TB cases, with a trough in December and peaks in July and October, but there was no significant seasonality. Temperature variations did not show a clear pattern with TB diagnoses. Rainfall alternated with TB diagnoses in the first half of the year, but then overlapped in the second half and was significantly associated with TB diagnoses. Influenza cases were significantly related to TB diagnoses with (β = 0.05, 95% CI 0.01 to 0.09, p = 0.01) or without (β = 0.06, 95% CI 0.01 to 0.1, p = 0.01) rainfall, and had particular overlap with pulmonary TB cases.

**Conclusions:**

Seasonal variations in childhood TB diagnoses were non-significant. Temperature did not have a clear pattern with TB diagnoses, but rainfall and influenza cases correlated with the primarily clinically diagnosed childhood TB cases.

## Introduction

Seasonality in tuberculosis (TB) has been described in non-equatorial regions, with greater cases in spring and early summer and troughs in late fall and winter [1–4]. The causes of this phenomenon are unclear [1, 2], and are grouped into factors that increase transmission (crowding in winter, humidity influencing survival of *Mycobacterium tuberculosis*), increase risk of progression (low vitamin D levels in winter [4, 5], air pollution [6], respiratory viral infections [2]), or delays in care such as during a holiday season [2].

One of the strongest arguments for increased transmission is that pediatric TB cases are more likely to have a seasonal component [2, 3, 7, 8]. In the United States, children less than 5 years had almost four times the seasonality compared to older ages [3]. In China and India, children 0–14 years had the highest seasonal variation [8, 9]. As opposed to adults where progression from exposure to disease can take years, children can develop primary disease soon after infection [10].

Past studies have not evaluated the seasonality of childhood TB in equatorial regions, where there are not four distinct seasons, but rather variations in wet and dry seasons. There are mixed results on the effects of latitude on TB, and there are few studies actually conducted in an equatorial country [2, 3, 11, 12]. An assessment of seasonality of childhood TB is important for elucidating drivers for TB seasonality, and can guide resource allocation, clinical evaluation and research enrollment in the many high TB burden countries at the equator. Our objectives were to assess the seasonality of childhood TB diagnoses over five years at a pediatric TB clinic in Kampala, Uganda, and compare trends in TB diagnoses to changes in environmental temperature, rainfall, and influenza cases.

## Materials and methods

### Setting

The study assessed children under 15 years old who presented for care at the Mulago National Referral Hospital outpatient pediatric TB unit in Kampala, Uganda. This clinic is the diagnostic and treatment unit of children with TB at the hospital. The diagnosis is largely based on clinical signs and symptoms, with review of a chest X-ray if available, and Xpert MTB/RIF testing on sputum samples if able to be performed, according to national guidelines [13]. The clinic operates throughout the year, except during late December and early January when it is closed for 2–3 weeks.

Kampala is the capital city, and is located at 0.34 degrees latitude at an elevation of 1,190 meters above sea level. The city has a tropical climate with average temperatures ranging from 21.7 °C to 23.9 °C [4]. Traditionally, there are two rainy seasons, a “long rain” from March to June and a “short rain” from September to November [14]. These generally correspond with influenza seasonality, with a major peak from September to November and smaller peak from March to June [15]. Influenza vaccine is not part of the routine immunization schedule for children [16].

The study was approved by the Mulago Hospital Research and Ethics Committee, and all data was de-identified prior to analysis.

### Data sources

We assessed retrospective clinical data from children started on anti-TB therapy at the Mulago Pediatric TB Unit during the period January 2010 through November 2015 (71 months). We extracted the data from an electronic database, including age, weight, TB symptoms, HIV status, tuberculin skin testing (TST) result (positive if ≥ 10 mm or ≥ 5 mm if HIV-infected), chest x-ray (abnormal or normal interpretation), case criteria (microbiologically confirmed by Xpert MTB/RIF testing or clinically diagnosed), type (pulmonary or extrapulmonary TB), and date of diagnosis. Data available in supporting information (S1 and S2 Files).

Average monthly temperature and precipitation data in Uganda from 2010 to 2015 was obtained from the World Bank [17]. Data on monthly influenza cases from 2010 to 2014 in Uganda were available from a hospital-based surveillance study at five government sites (including Mulago National Referral Hospital) of adults and children with influenza-like-illness, and influenza A or B confirmed by real-time polymerase chain reaction (RT-PCR) [15].

### Statistical approach

We used descriptive statistics of frequency, proportion, median and interquartile range (IQR) to summarize the data. We aggregated case counts into monthly time series for seasonality analysis. All case types and criteria were included; the limited extra-pulmonary and confirmed cases did not allow for stratified analysis.

Seasonality was defined as an intra-year variation that is stable at predictable intervals. To assess seasonality of TB, we used the X-13ARIMA-SEATS seasonal adjustment program from the United States Census Bureau to decompose the observed monthly time series (X_t_) into its trend (T_t_), seasonal (S_t_) and remainder or irregular (I_t_) components [3, 18–20]. In this approach, a symmetric moving average was applied to the time series to estimate the trend. Using an additive model (X_t_ = T_t_ + S_t_ + I_t_), the time series was then de-trended by subtracting the trend from the observed time series. The seasonal component was determined by weighted moving averages of each month of the de-trended series. The irregular component was created by subtracting the trend and seasonal components, and was assessed for outliers. These steps were then repeated iteratively to obtain a final estimation of the trend, seasonal and irregular components.

The mean annual seasonal amplitude was calculated from the seasonal component as the annual difference between the peak and trough as a proportion of the annual mean case count. Seasonality was determined using the F test and Kruskal-Wallis tests for stable seasonality (significance defined as less than the 0.1 percent and 1 percent level, respectively), the F test for moving seasonality (significance defined at the 5 percent level), and the combined test for identifiable seasonality that assesses all three tests.

We plotted the mean TB cases by month over the monthly mean temperature (in degrees Celsius), rain (in centimeters), and influenza cases to assess overlapping patterns. Multivariate Poisson regression with log link and robust standard errors was used to evaluate the association of temperature, rainfall, and influenza cases on TB case counts. Interaction terms between temperature, rainfall and influenza were evaluated and included if significant (p < 0.05). Coefficients (β) with 95% confidence intervals (CIs) were presented, with significance defined as p < 0.05.

We performed analyses using R version 3.5.1 (www.r-project.org/), in particular the *seasonal*[21], *astsa*[22], and *forecast*[23] packages, and STATA 15 (StataCorp, College Station, Texas, USA).

## Results

### Participant characteristics

There were 713 children diagnosed with TB from January 2010 to November 2015 (Table 1). The median age was 37 months (IQR 16–84), with almost a quarter under 5 years old. HIV testing was available for 98% of children, and 47 (7%) were HIV positive. Over two-thirds of children (n = 492, 69%) had pulmonary TB, and 609 (85%) were clinically diagnosed. The majority of children confirmed or clinically diagnosed TB had an abnormal chest X-ray (N = 598/671, 89%); less than half had a TST performed, of which almost three quarters were positive (N = 236/322, 73%). Of those with clinically diagnosed TB, review of available data showed that 495 (81%) met clinical criteria for presumptive TB [13]; 30 (5%) had an abnormal CXR and 43 (7%) had physical signs concerning for TB (pulmonary or extra-pulmonary). Extra-pulmonary cases included adenitis, spinal TB and meningitis.

**Table 1:** Child tuberculosis cases at Mulago Hospital, Kampala, Uganda, 2010–2015.

Characteristic	N (%)^1^
Age group (N = 712)	
<5 yrs	173 (24)
5–9 yrs	439 (62)
>9 yrs	100 (14)
Female (N = 713)	323 (45)
BCG vaccinated^2^ (N = 475)	281 (59)
HIV positive (N = 701)	47 (7)
Underweight (Weight-for-Age Z score ≤ -2) (N = 386)	169 (44)
Tuberculin Skin Test Positive^3^ (N = 322)	236 (73)
Chest X-ray performed (N = 671)	598 (89)
Abnormal (N = 489)	456 (93)
Pulmonary TB (N = 711)	492 (69)
Extra-pulmonary TB (N = 711)	219 (31)
Type (N = 213)	
Adenitis	80 (38)
Spinal	49 (23)
Meningitis	25 (12)
Abdominal	29 (14)
Disseminated	19 (9)
Pericarditis	4 (2)
Pleural	7 (3)
Confirmed TB (N = 673)	64 (10)
Clinical TB (N = 673)	609 (90)
Cough (N = 533)	415 (78)
Fever (N = 527)	373 (71)
Failure to Thrive (N = 418)	172 (41)
Weight Loss (N = 481)	287 (60)
Chest Pain (N = 408)	43 (11)
Loss of Playfulness (N = 471)	199 (43)
Lymphadenopathy (N = 482)	144 (30)

BCG: Bacillus Calmette–Guérin; HIV: Human Immunodeficiency Virus; TB: tuberculosis

^1.^ Total N noted with each characteristic

^2.^ Defined by report or presence of scar

^3.^ Defined as ≥ 10 mm or ≥ 5 mm if HIV positive

### Seasonality

**Fig 1:**
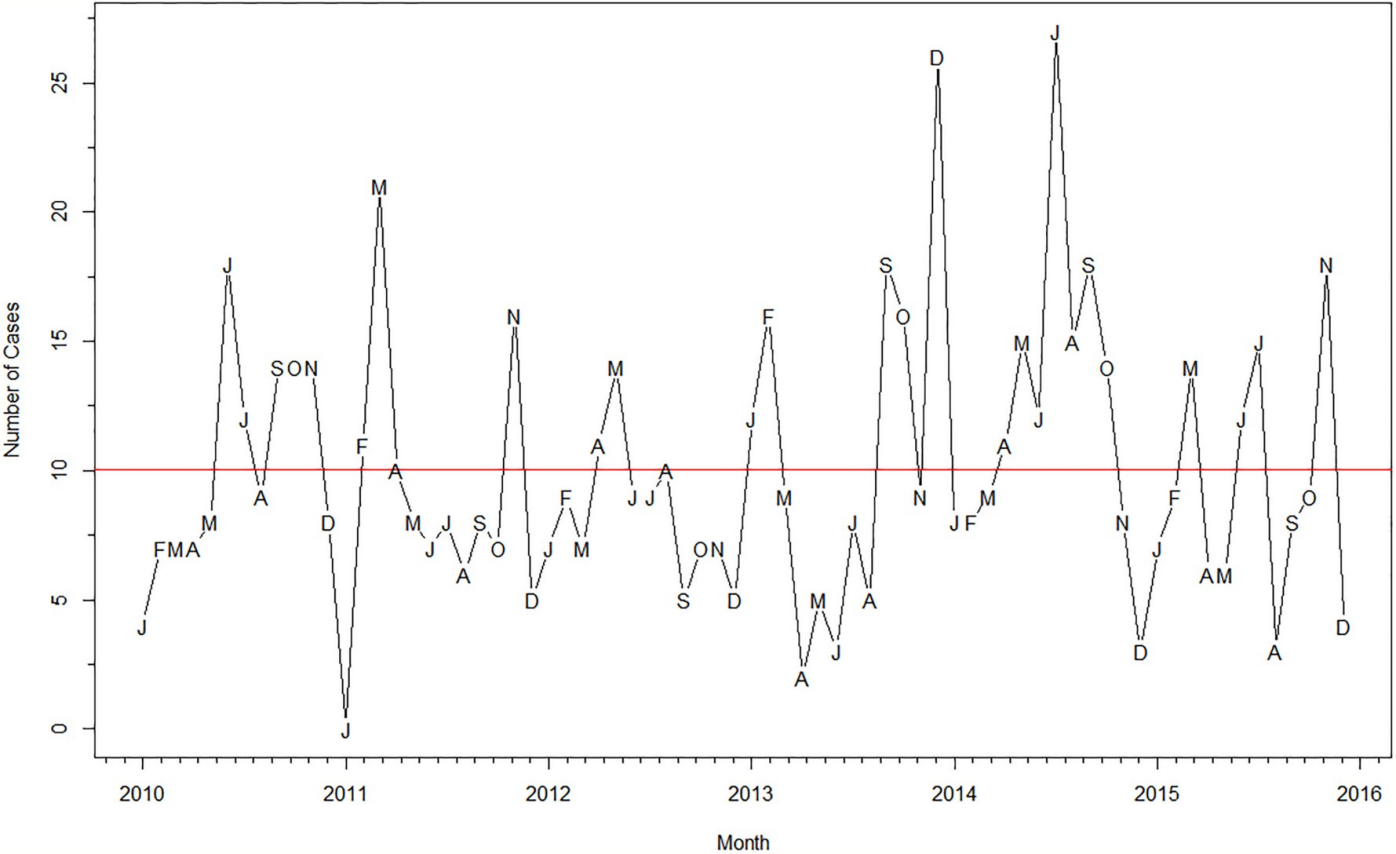
Time series of pediatric TB cases, Kampala, Uganda, 2010-2015. Months indicated by letters, red line represents the mean number of cases per month (10) over the five-year period.

**Fig 2:**
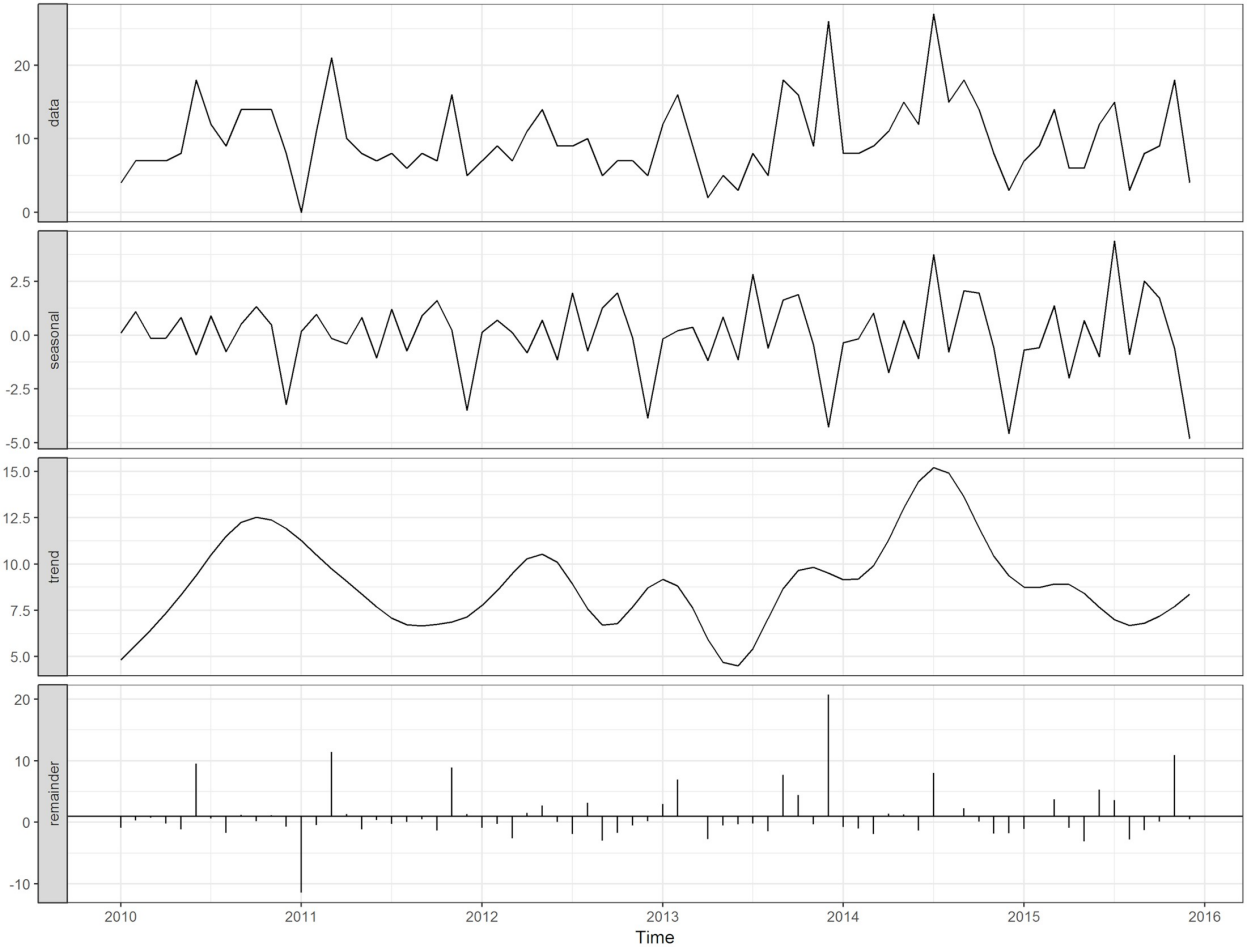
Time series decomposition of pediatric TB cases, Kampala, Uganda, 2010–2015.

**Fig 3:**
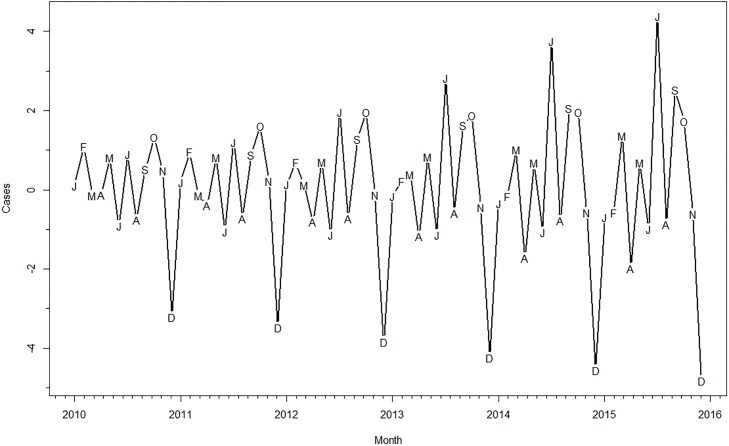
Seasonal component of pediatric TB cases, Kampala, Uganda, 2010–2015. Months indicated by letters.

Fig 1 shows the monthly time series of pediatric TB diagnoses from January 2010 to November 2015. There were a mean 10 cases per month during this period, with minimum cases (0) diagnosed in January 2011 and a maximum of 27 cases diagnosed in July 2014. Fig 2 shows the decomposition of the TB cases into its seasonal, trend and remainder components. The seasonal component (Fig 3) and corresponding seasonal adjustment factors (S1 Table) suggested a trough in December and peaks in July, September and October. The mean annual seasonal amplitude was 66%, although it reflected small changes (range -5 to +4 cases). The final model was an ARIMA (1 0 2), with non-seasonal AR (1) estimate -0.99 (SE 0.04), non-seasonal MA (1) estimate of -1.38 (SE 0.12), and MA (2) estimate of -0.43 (SE 0.11). The F-test for stable seasonality and for moving seasonality were non-significant at the 0.1 percent and 5 percent level, respectively. The Kruskal-Wallis test did have evidence of seasonality at the 1 percent level. Combining all three, however, there was no identifiable seasonality.

### Role of temperature, rain and influenza cases on TB diagnoses

**Fig 4:**
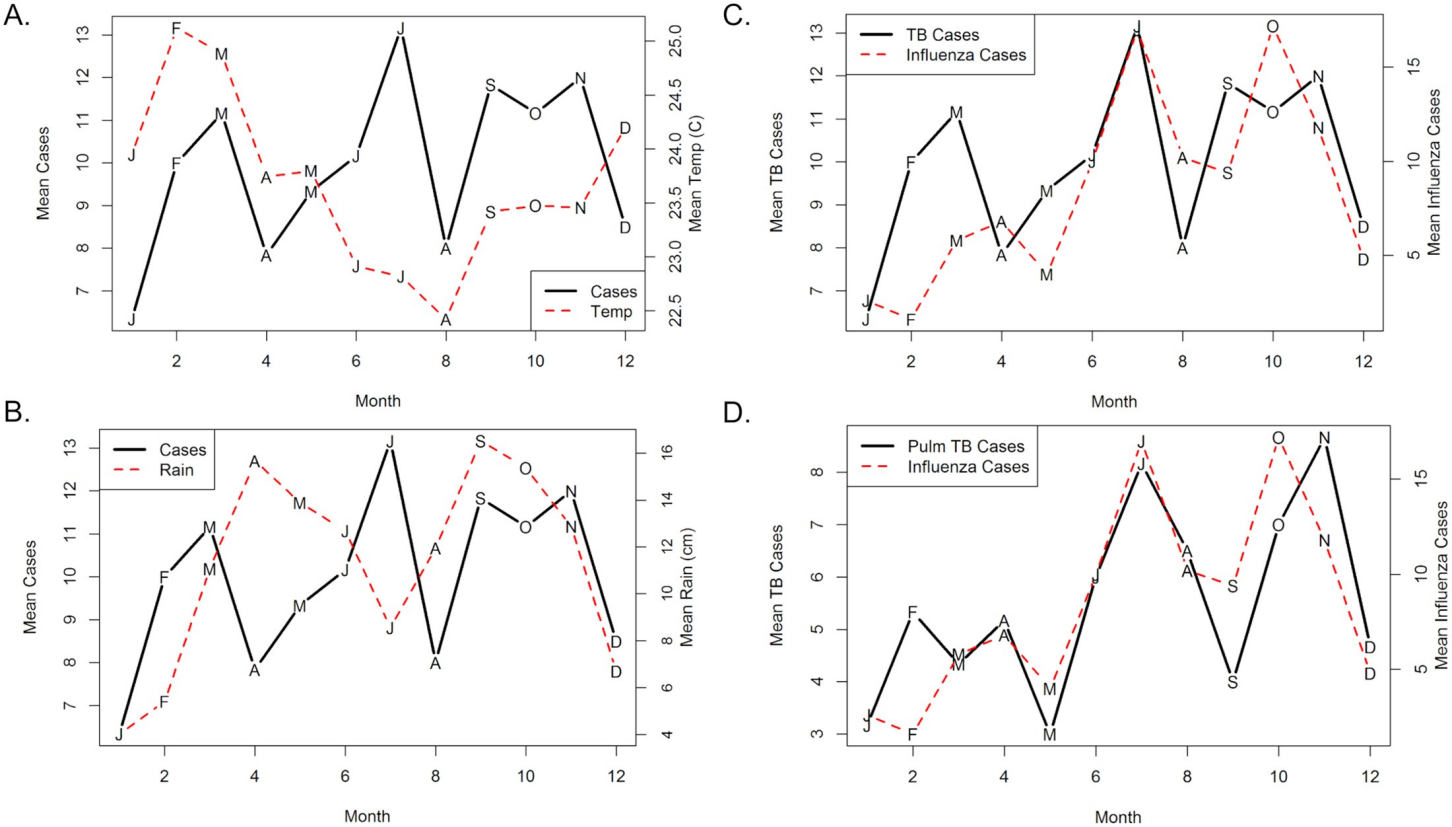
Monthly mean plots of pediatric TB cases vs. Temperature, rainfall and influenza cases. (A) Pediatric TB Cases vs. Temperature. Months indicated by letters. (B) Pediatric TB Cases vs. Rainfall. (C) Pediatric TB Cases vs. Influenza Cases. Data on influenza cases available from 2010–2014. (D) Pediatric Pulmonary TB Cases vs. Influenza Cases.

Plots of monthly mean pediatric TB cases compared to monthly mean temperature, rain and influenza cases are displayed in Fig 4. The temperature range was narrow (2.7 °C) and there was no clear relationship with TB cases (Fig 4A). Peaks of rain appeared to alternate with peaks in TB cases in the first half of the year, but then overlapped with TB case peaks in September, October and November (Fig 4B). Comparison of influenza cases to TB cases showed overlap in the second half of the year (Fig 4C). When only pulmonary cases were included (Fig 4D), the curves had notable overlap throughout the year. Multivariate Poisson regression further revealed that rainfall and influenza cases, but not temperature, were significantly associated with TB cases (Table 2). There was also a small but significant interaction of rain and influenza.

**Table 2:** Multivariate poisson regression of temperature, rain and influenza on pediatric TB cases, Kampala, Uganda, 2010–2014^1^.

Variable	β	95% CI	P-Value
Temperature (°C)	0.08	-0.03 to 0.20	0.14
Rain (cm)			
Without Influenza	0.04	0.01 to 0.07	0.02
With Influenza^2^	0.04	0.004 to 0.07	0.03
Influenza Case			
Without Rain	0.06	0.01 to 0.1	0.01
With Rain^2^	0.05	0.01 to 0.09	0.01

^1.^ Influenza data did not include 2015

^2.^ The interaction term for rain and influenza was significant (β = -0.004, 95% CI -0.01 to -0.0006, p = 0.02). Consequently, we present rain and influenza with and without this interaction.

## Discussion

At a national referral hospital in Kampala, Uganda, we found peaks of childhood TB diagnoses in July, September and October, and troughs in December from 2010 to 2015, with an average 66% annual seasonal amplitude. However, the absolute changes were small and overall were not statistically significant. Rain peaks overlapped with TB case peaks in the second half of the year, and rises in influenza cases were significantly associated with greater childhood TB diagnoses. This is the first known assessment of the seasonality of childhood TB in an equatorial setting.

Evidence of TB seasonality at the equator has been equivocal. Given relatively stable weather and sunshine, it is hypothesized that there would be less variability in crowding and thus less seasonal changes [5, 12, 24]. Studies have tested this by stratifying seasonality by latitude. Evaluations in Australia and India found that regions closer to the equator did not show seasonality while areas farther from the equator had seasonality [11, 12]. But an analysis in the United States found no difference in seasonality stratified by latitude [3]. There are few studies on seasonality in actual equatorial regions and none assessed childhood TB [25–27]. A study in Singapore found seasonal peaks similarly in July and October, and also noted that they were small absolute changes [27]. Our results support there is limited seasonality among childhood TB cases at the equator.

The trough in December likely reflects reduced health-seeking behavior and clinic closure for part of the month in the holiday period. The temperature range in Uganda was narrow and did not show a relationship with cases. Rainfall had an alternating pattern in the beginning of the year, which may reflect more crowding followed by progression to disease. Yet, we also found overlap of rainfall with TB cases later in the year and a positive association in regression analysis. The role of rain on TB has been mixed; studies in Cameroon, Benin, Tanzania and China found higher cases during the wet season and with greater rainfall, in Nigeria there was no significant difference, and in Iran the areas with low rainfall had the higher TB incidence [25, 26, 28–31]. The mechanism of any relationship is unclear, with prior studies suggesting greater survivability of *M*. *tuberculosis*, concomitant respiratory illnesses, or lower vitamin D levels in the rainy season [2, 31]. As opposed to prolonged periods of cold weather that promote indoor behavior, duration and severity of rainy seasons can vary from year to year, and may not rain the entire day [32]. The weather in East Africa is also changing and rainfall has been lower than predicted, known as the East Africa Climate Paradox [33]. Rain may also affect the ability to come for evaluation; we found a small but significant reduction in the relationship of influenza on TB diagnoses with greater rain. The role of rain on childhood TB diagnoses in Uganda is likely multi-factorial and requires further evaluation.

Seasonality in childhood TB has been largely attributed to increased transmission from crowding in colder months [8, 34]. In our setting, we did not find a relationship between temperature and TB diagnoses, but we noted a significant association with influenza. There are several possibilities for this relationship. During the influenza season, there may be greater evaluation of respiratory diseases including TB and thus more diagnoses [34]. Given paucibacillary disease in children, the majority of TB diagnoses were based on clinical symptoms and chest X-ray. We found that pulmonary TB in particular overlapped with influenza cases; the similar presentation may have led to misdiagnoses for influenza or other respiratory viral illnesses. At the same time, animal models have found influenza co-infection can reduce mycobacterial clearance and increase mortality by increasing type I interferon, reducing T cell responses, and lowering MHC expression [35, 36]. In South Africa, influenza co-infection was associated with higher TB mortality [37], and more children were hospitalized with TB following influenza peaks, with the authors suggesting that influenza may have promoted adult TB reactivation and subsequent infection in children [38]. Thus, while the relationship between influenza and childhood TB may be partly due to misdiagnosis, there is also epidemiologic and biologic plausibility that more cases were being detected due to more evaluation, higher risk of infection and progression, and greater severity of disease during the influenza season.

Evaluation of TB seasonality often relies on national surveillance data, which frequently lacks individual-level details. By accessing records at a pediatric TB clinic, we were able to better characterize the TB cases and criteria for diagnosis. There were also several limitations to our analysis. Non-significant seasonality could have been due to the low number of cases per month. This also prevented stratification by pulmonary vs. extra-pulmonary and clinical vs. confirmed cases. Since the majority of cases were clinically diagnosed, this may not reflect true TB case patterns. However, data was available on 93% of clinically diagnosed cases to support that national guidelines for child TB diagnosis were followed. Available rain and temperature data were not specific to Kampala and represented national averages, limiting direct comparison. Adults were included in the influenza cases, but this data served to show the overlapping influenza season rather than confirmed TB-influenza coinfection. Influenza cases were identified from hospital-based surveillance and may suggest periods of severe influenza requiring hospitalization rather than the full season.

Of the 30 high burden TB countries, over half are at or near the equator. We provide one of the few studies on seasonality in the region, and the only one focused on children. While children are often used as a proxy for TB transmission, it is important to recognize that other factors may influence their trends and seasonality, such as influenza infection. Further studies are needed in equatorial countries to understand any seasonal patterns and associated factors for planning the appropriate care, public health interventions and research to reduce the burden of disease.

## Supporting information

S1 Table Seasonal adjustment factors. (DOCX)

S1 File Mulago paediatric TB Unit Dataset, 2010–2015. (CSV)

S2 File Mulago paediatric TB Unit monthly time series with rainfall, temperature and influenza cases, 2010–2015. (CSV)
