# Supplementary material for: Seasonality of childhood tuberculosis cases in Kampala, Uganda, 2010-2015
Source: PLoS One. 2019 Apr 9;14(4):e0214555. doi: 10.1371/journal.pone.0214555 (PMC6456174; doi:10.1371/journal.pone.0214555)
Supplement: S1 Table — (DOCX) [file pone.0214555.s001.docx]

**S1 Table. Seasonal Adjustment Factors**

| **Year** | **Jan** | **Feb** | **Mar** | **Apr** | **May** | **Jun** | **Jul** | **Aug** | **Sep** | **Oct** | **Nov** | **Dec** | **Amp^1^** |
| --- | --- | --- | --- | --- | --- | --- | --- | --- | --- | --- | --- | --- | --- |
| **2010** | 0 | 1 | 0 | 0 | 1 | -1 | 1 | -1 | 1 | 1 | 0 | -3 | 0.39 |
| **2011** | 0 | 1 | 0 | 0 | 1 | -1 | 1 | -1 | 1 | 2 | 0 | -3 | 0.56 |
| **2012** | 0 | 1 | 0 | -1 | 1 | -1 | 2 | -1 | 1 | 2 | 0 | -4 | 0.72 |
| **2013** | 0 | 0 | 0 | -1 | 1 | -1 | 3 | -1 | 2 | 2 | 0 | -4 | 0.65 |
| **2014** | 0 | 0 | 1 | -2 | 1 | -1 | 4 | -1 | 2 | 2 | -1 | -5 | 0.73 |
| **2015** | -1 | -1 | 1 | -2 | 1 | -1 | 4 | -1 | 3 | 2 | -1 | -5 | 0.92 |
| **Mean** | -0.17 | 0.33 | 0.33 | -1.0 | 1.0 | -1.0 | 2.5 | -1.0 | 1.67 | 1.83 | -0.33 | -4.0 | 0.66 |

1. The Annual Seasonal Amplitude, defined as the difference in peak and trough divided by the mean case count for the year
